# Predictors of Racial Differences in Depression and Affect among Older adult, Long-term Cancer Survivors

**DOI:** 10.1093/geroni/igab046.2582

**Published:** 2021-12-17

**Authors:** Gary Deimling, Dyanna Burnham, Gabrielle Beck

**Affiliations:** 1 Case Western Reserve University, Cleveland, Ohio, United States; 2 Case Western Reserve University, CLeveland, Ohio, United States

## Abstract

Research has long documented the psycho-social sequelae experienced by those who have been treated for and survived cancer. Depression, affect and other indicators of mood state have been an important focus of that research However, there is little research on racial differences in depression and affect outcomes or the specific cancer and age-related factors that predict them. The research to be presented is based on a 10 year, six wave NCI funded study of 471 older adult (age 60+), long-term cancer survivors randomly selected from the tumor registry of a comprehensive cancer treatment center. Key outcome measures were depression (CES-D) scale) and both positive and negative affect (PANAS). Covariance analyses and nested OLS Regression were used to identify Black-white differences these outcomes and the relative importance of both cancer and non-cancer predictors. Blacks reported lower levels of depression and negative affect when compared to whites. In a separate regression analysis of the black sub-sample, continuing cancer-related symptoms were by far the strongest predictors (beta =.16) of negative affect. In the white sub-sample, while cancer-related symptoms continued to be a significant predictor (beta=.16), non-cancer symptoms were substantially more important (beta=. 22). These results will hopefully help practitioners to have a better understanding of the nuanced racialized experiences and mental health among cancer survivors, and how these may impact after-care for older adult cancer survivors.

